# Endothelial activation impairs the function of small extracellular vesicles

**DOI:** 10.3389/fphar.2023.1143888

**Published:** 2023-03-27

**Authors:** Nicolas Herrera-Zelada, Úrsula Zúñiga-Cuevas, Andrés Ramírez-Reyes, Ignacio Norambuena-Soto, Leslye Venegas-Zamora, Mayarling F. Troncoso, Alejandra Hernández, Gina Sánchez, Zully Pedrozo, Sergio Lavandero, Jaime A. Riquelme

**Affiliations:** ^1^ Advanced Center for Chronic Disease (ACCDiS), Facultad de Ciencias Químicas y Farmacéuticas & Facultad de Medicina, Universidad de Chile, Santiago, Chile; ^2^ Programa de Fisiopatología, Instituto de Ciencias Biomédicas (ICBM), Facultad de Medicina, Universidad de Chile, Santiago, Chile; ^3^ Programa de Fisiología y Biofísica, ICBM, Facultad de Medicina, Universidad de Chile, Santiago, Chile; ^4^ Department of Internal Medicine (Cardiology Division), University of Texas Southwestern Medical Center, Dallas, TX, United States; ^5^ Departamento de Química Farmacológica y Toxicológica, Facultad de Ciencias Químicas y Farmacéuticas, Universidad de Chile, Santiago, Chile; ^6^ Interuniversity Center for Healthy Aging, Santiago, Chile

**Keywords:** endothelial cells (ECs), small extracellular vesicles (sEVs), ischemia/reperfusion (I/R), cardioprotection, endothelial activation

## Abstract

Small extracellular vesicles are nanosized vesicles (30–200 nm) that can ferry proteins, nucleic acids, and lipids between cells and therefore, have significant potential as biomarkers, drug delivery tools or therapeutic agents. SEVs of endothelial origin have been shown to -among other functions-reduce *in vitro* ischemia/reperfusion (I/R) injury in cardiomyocytes, but whether a pro-inflammatory state of the endothelium impairs the functionality of these SEVs remains to be elucidated. To test this, human umbilical vein endothelial cells cells were treated with TNF-α 10 ng/mL and the expression of the pro-inflammatory parameters VCAM-1, ICAM-1 and eNOS were determined by Western blot. SEVs were isolated from endothelial cells treated with or without TNF-α 10 ng/mL using size exclusion chromatography. The size and concentration of SEVs was measured by Nanoparticle Tracking Analysis. The expression of the surface marker CD81 was determined by immunoassay, whereas their morphology was assessed by electron microscopy. The function of endothelial SEVs was assessed by evaluating their cardioprotective effect in an *ex vivo* model of global I/R using isolated hearts from adult C57BL/6 mice. Treatment of HUVECs with TNF-α induced the expression of VCAM-1 and ICAM-1, whereas eNOS levels were decreased. TNF-α did not affect the production, size, morphology, or expression of CD81. SEVs significantly reduced the infarct size as compared with untreated mice hearts, but SEVs isolated from TNF-α treated cells were unable to achieve this effect. Therefore, a pro-inflammatory state induced by TNF-α does not alter the production of endothelial SEVs but impairs their function in the setting of I/R injury.

## Introduction

Small extracellular vesicles (SEVs) -also known as exosomes-are critical mediators of cellular communication, and in recent years, these nanosized vesicles (30–200 nm) have gained significant attention due to their role in the transport of lipids, nucleic acids, and proteins from 1 cell to another, thereby mediating intercellular communication (Zhao et al., 2021). SEVs produced by the endothelium -a monolayer of cells that regulates vascular tone, permeability, inflammation, coagulation, and angiogenesis (Kruger-Genge et al., 2019)- have been proposed to be potential biomarkers or even used as drug delivery carriers to treat a dysfunctional endothelium (Desideri et al., 2021). Furthermore, previous studies suggest endothelial SEVs may be promising therapeutic agents in the context of cardiac ischemia/reperfusion (I/R) injury, whereby these nanovesicles have been shown to reduce death of adult rat cardiomyocytes subjected to hypoxia/reoxygenation (Davidson et al., 2018a; Davidson et al., 2018b). However, the cargo and function of endothelial SEVs may vary depending on multiple factors, such as cell type, redox state, or pro-inflammatory conditions (Desideri et al., 2021). When the endothelium is exposed to inflammatory mediators, it develops endothelial activation, which is manifested by a pro-coagulant and pro-inflammatory state (Pober, 2002; Hromada et al., 2017). Furthermore, it has been reported that pro-inflammatory stimulus can trigger an increased production of endothelial SEVs (Li et al., 2019) and increase the content of inflammatory mediators carried by these nano-sized vesicles (Hosseinkhani et al., 2018), but whether these SEVs remain functional, especially regarding their therapeutic effect, remains poorly explored. Thus, we sought to evaluate whether induction of a pro-inflammatory state can impair the function of endothelial-derived SEVs, by testing their cardioprotective effect in I/R.

## Methodology

### Animals

All animal experiments were performed in accordance with the Guide for the Care and Use of Laboratory Animals by the U.S. National Institutes of Health (NIH Publication, eighth Edition, 2011). The protocol was approved by the Institutional Ethics Review Committee, Universidad de Chile, code: CBE 2018–16.

### Cell culture

Primary human umbilical vein endothelial cells (HUVEC) were acquired from Lonza and maintained in Endothelial Basal Medium-2 (Lonza) and supplemented with EGM-2 SingleQuot kit (Lonza). Cells were used between passages 3–12 and were incubated at 37°C and 5% CO_2_. HUVECs were treated with TNF-α (10 ng/mL) for 24 h or 48 h, followed by the different experimental approaches detailed below.

### Western blot

Proteins were separated from cell lysates *via* SDS-PAGE, using 8% polyacrylamide gels and proteins were then transferred onto PVDF membranes *via* wet transfer. Membranes were incubated with primary antibodies for Intercellular Adhesion Molecule 1 (ICAM-1) (sc 13160 - Santa Cruz, mouse, 1:1000), Vascular Adhesion Molecule 1 (VCAM-1) (sc-13160 Santa Cruz, mouse, 1:1000) and endothelial nitric oxide synthase (eNOS) (610297 - BD, mouse, 1:1000) using β-Tubulin (T-0198 - Sigma, mouse, 1:5000) as a loading control. The intensity of horseradish peroxidase conjugated secondary antibodies (402335 - anti-mouse 1:5000, Calbiochem, 401315 - rabbit 1:5000, Calbiochem) was acquired using Odyssey Fc (LiCor Biosciences), and quantification of bands was performed *via* densitometry analysis using the UN-SCAN-IT software.

### Determination of soluble Interleukin-6

The supernatant of HUVECs treated with or without TNF-α for 24 h was collected and assessment of soluble Interleukin-6 (sIL-6) protein levels was determined by performing an ELISA assay using a kit (R&D Systems), according to the manufacturer’s instructions.

### Immunofluorescence

HUVECs were cultured on coverslips, fixed with 4% paraformaldehyde w/v and permeabilized with 0.1% Triton X-100. Cells were stained with primary antibodies: RAB7 (9367, Cell signalling Technology) and CD63 (556019, BD Bioscience), and secondary antibodies: Goat anti-Rabbit IgG (H + L) Highly Cross-Adsorbed Secondary Antibody, Alexa Fluor™ 488 (A-11034, Invitrogen) for Rab7; and Goat anti-Mouse IgG (H + L) Highly Cross-Adsorbed Secondary Antibody, Alexa Fluor™ 568 (A-11031, Invitrogen) for CD63. The nuclei were stained using Hoechst 33258 (10 μg/mL). Images were obtained using a fluorescence microscope (ZOETM, BioRad) and colocalization was determined by calculation of Mander’s Coefficient.

### Isolation and characterization of SEVs

To isolate endothelial SEVs, 3 T-75 flasks per condition of HUVECs, with 80%–90% confluence were used. Cells were pre-treated with vehicle (phosphate-buffered saline) or TNF-α (10 ng/mL) for 24 h. Then, conditioned medium was removed and replaced with a medium containing 2% of exosome-depleted serum and treated again with or without TNF-α for an additional 24 h. The pre-treatment step was performed to ensure all SEVs isolated from TNF-α-treated HUVECs were isolated from cells in a pro-inflammatory state. To isolate SEVs after the indicated treatments, cell medium was collected and centrifuged at 300 *g* (10 min, 4°C), 2000 g (10 min, 4°C) and 10000 g (30 min, 4°C). Samples were concentrated by centrifugation of 10 kDa Amicon Filters (Merck) at 5000 g for 90 min. Then, SEVs were isolated using size exclusion chromatography (qEV-IZON). Quantification of particle concentration and measurement of modal size was performed using Nanoparticle Tracking Analysis (NTA) on a NanoSight NS300 (Malvern). Assessment of CD81 expression in SEVs was determined by an ELISA assay, using a kit (CSB-EL004960HU, Cusabio), according to the manufacturer’s instructions. The morphology of SEVs was evaluated using a *Uranyless* staining protocol and images were obtained with by Electron Microscopy using Talos F200C G2 microscope.

### *Ex vivo* ischemia/reperfusion

For the functional assessment of SEVs, 8–12 weeks old male C57BL/6J mice were anesthetized with pentobarbital 60 mg/kg + heparin 100 IU intraperitoneally. After anesthesia, the hearts were quickly extracted, cannulated *via* the aorta and retrogradely perfused with a Krebs-Henseleit buffer in a Langendorff system at 37°C, as previously described (Rossello et al., 2017), but using constant flow perfusion. The hearts were stabilized for 20 min and then, 10^8^ particles/mL of SEVs were administered for 10 min, followed by 35 min of global ischemia and 120 min of reperfusion. Perfusion pressure was recorded continuously throughout the experimental period using PowerLab software (ML866 AD Instruments, Australia).

### Infarct size measurement

At the end of reperfusion, hearts were removed from the Langendorff rig and perfused with 5 mL of 1% 2, 3, 5-triphenyltetrazolium chloride (Sigma) in PBS pH 7.4 at 37 °C for 10 min, to establish the viable (red) and the infarcted (white) myocardium. Then, hearts were frozen at −20°C for 1 h and were later cut into five slices, which were then fixed using a 4% w/v paraformaldehyde solution at room temperature. Images were obtained and a planimetry analysis using ImageJ software (NIH, Bethesda, MD, USA) was performed by a blinded operator.

### Statistical analysis

For comparison of two groups, the Mann-Whitney test was used and Kruskal–Wallis’s test with Dunn’s multiple comparison analysis for non-parametric data for multiple comparisons. Data were presented with mean ± SD. Significant differences were considered at *p* < 0.05.

## Results

**FIGURE 1:**
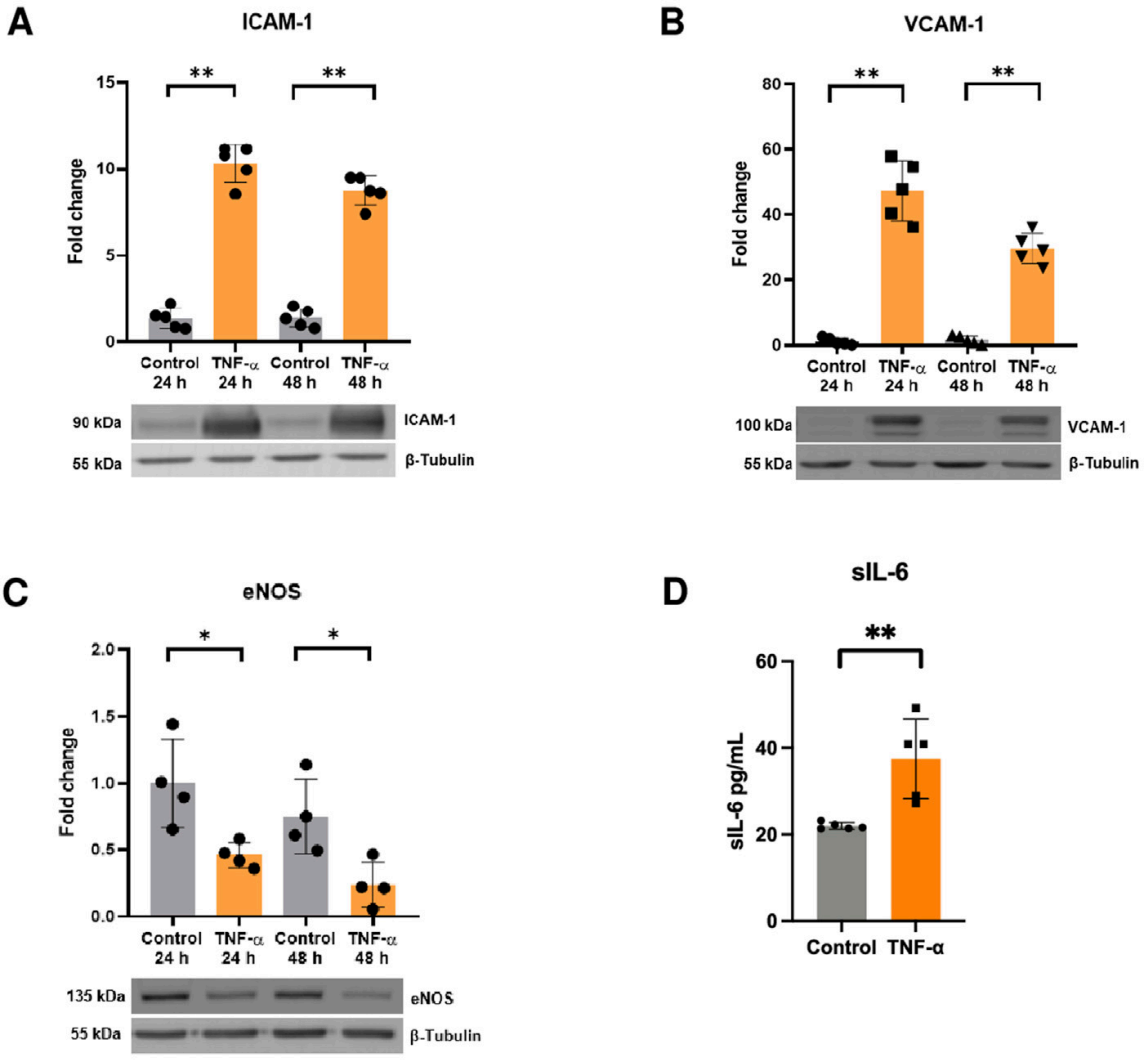
TNF-α induces endothelial activation. Protein levels of **(A)** ICAM-1 (n = 5), **(B)** VCAM-1 (n = 5), and **(C)** eNOS in response to TNF-α 10 mg/mL for 24 h and 48 h determined by Western blot (n = 4). **(D)** sIL-6 levels were assessed by ELISA after administration of TNF-α for 24 h (n = 5). *p < 0.05, **p < 0.01 vs. 24 h control, 48-h control, or control. Mean ± SD.

First, we aimed to establish the pro-inflammatory phenotype of endothelial cells in response to TNF-α. To achieve this, HUVECs were treated with TNF-α (10 ng/mL) for 24 and 48 h and the expression of pro-inflammatory markers was evaluated by Western blot. The results show that TNF-α induced the expression of ICAM-1, VCAM-1 and decreased the protein content of eNOS (Figures 1A–C). Moreover, TNF-α also increased the release of sIL-6 at 24 h (Figure 1D), thereby confirming the pro-inflammatory state of endothelial cells (TNF-α-activated endothelium).

**FIGURE 2:**
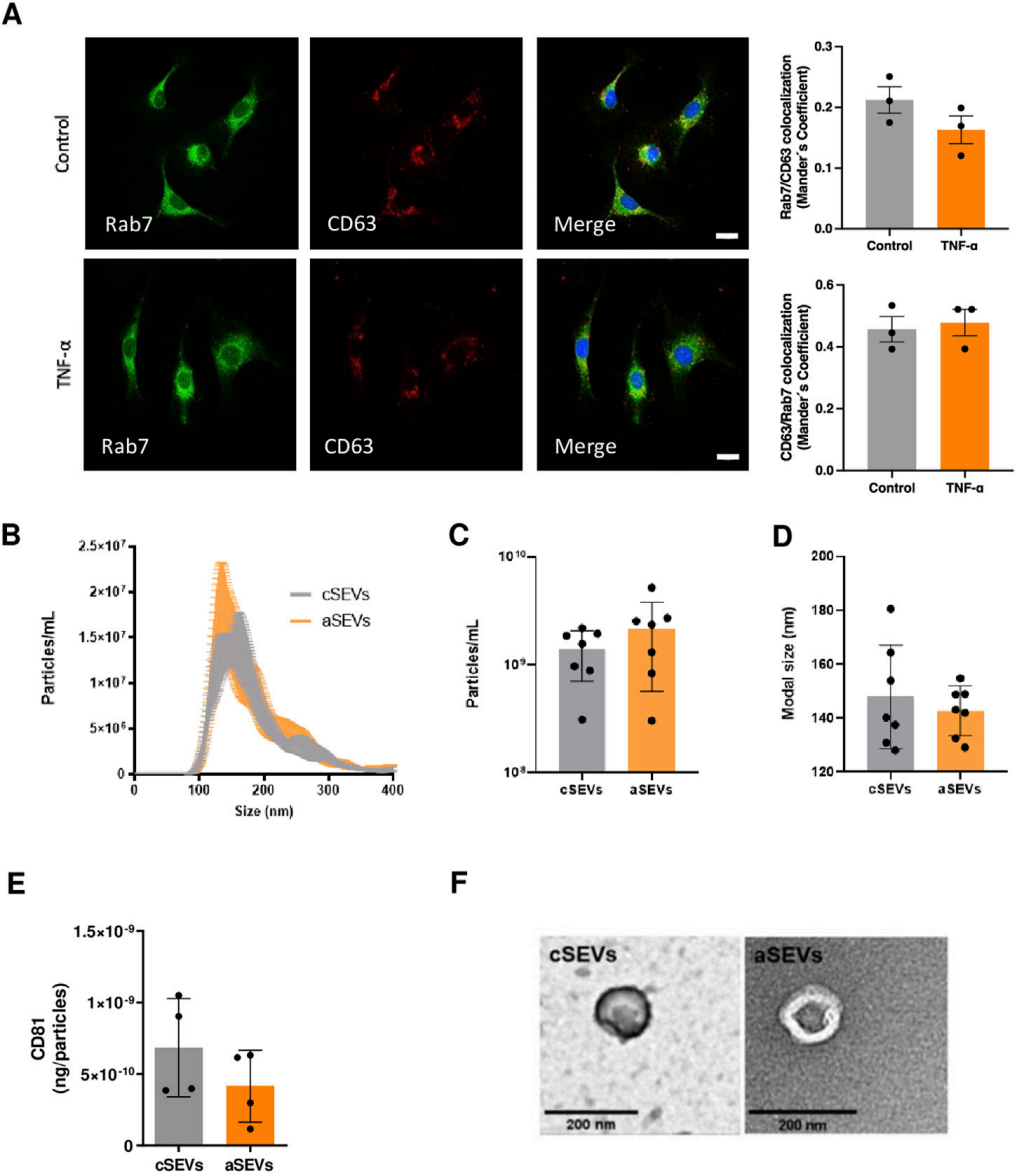
TNF-α does not affect production, expression of surface markers, size or morphology of endothelial-derived SEVs. **(A)** Immunofluorescence showing co-localization of Rab7 and CD63 in endothelial cells treated with or without TNF-α (n = 3) Scale bar: 20 μm. **(B)** Size distribution (n = 7), **(C)** concentration (n = 7), **(D)** modal size (n = 7) determined by Nanoparticle Tracking Analysis. **(E)** CD81 expression determined by ELISA (n = 4), and **(F)** morphology of endothelial small extracellular vesicles treated with (aSEVs) or without (cSEVs) TNF-α assessed by electronic microscopy. Scale bar: 200 nm. Mean ± SD.

Treatment of HUVECs with TNF-α (10 ng/mL) for 24 h did not increase the intracellular production of the SEV, evaluated by immunofluorescence and colocalization analysis of CD63 and the multivesicular body marker Rab7 (Figure 2A). This result may suggest that treatment with TNF-α had no effect on SEVs production. To confirm these findings, SEVs were isolated from both control endothelium (cSEVs) and TNF-α-activated endothelium (aSEVs). To achieve this, we pre-treated HUVECs with or without TNF-α for 24 h to ensure all SEVs derived from TNF-α-treated cells originated from cells in a pro-inflammatory state. After pre-treatment, cell medium was replaced, and cells were stimulated with TNF-α or vehicle (PBS) for an additional 24 h. SEVs were isolated using size exclusion chromatography (qEV-IZON) and quantification of particle concentration and measurement of modal size was performed using Nanoparticle Tracking Analysis (NTA). We observed that TNF-α did not increase the production or change the normal size of SEVs (Figures 2B–D), which was further confirmed by assessment of the expression of CD81 per particles in both cSEVs and aSEVs, using an ELISA assay and the NTA data. Indeed, TNF-α did not modify the expression of CD81 in endothelial SEVs (Figure 2E). Furthermore, both cSEVs and aSEVs have the typical “cup shaped” morphology of SEVs, as evaluated by Electron Microscopy (Figure 2F).

**FIGURE 3:**
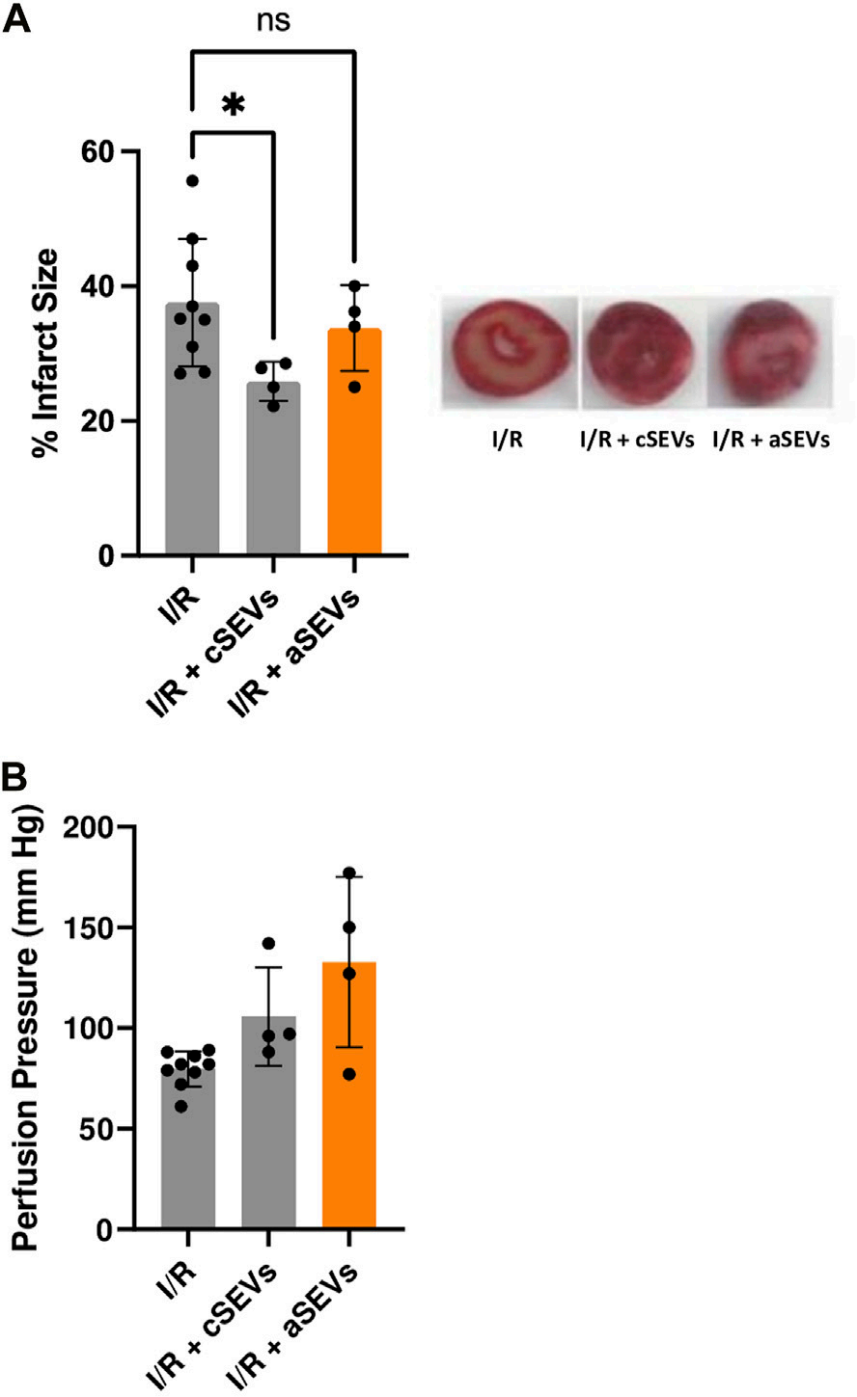
TNF-α impairs the function of endothelial SEVs in the setting of cardiac ischemia/reperfusion injury. **(A)** Infarct size with their respective representative images and **(B)** Perfusion pressure of isolated mice hearts treated with vehicle (n = 9), 1 × 10 ^8^ of cSEVs (n = 4) or aSEVs (n = 4) for 10 min before 35 min of global ischemia, followed by 120 min of reperfusion. I/R: ischemia/reperfusion, ns: not significant. *p < 0.05 vs. I/R. Mean ± SD.

To investigate whether endothelial activation affects the function of SEVs, we evaluated the cardioprotective effect of cSEVs and aSEVs in an *ex vivo* model of global I/R injury. To test this, hearts from adult C57BL/6J mice were isolated using a Langendorff system. After stabilization, 10^8^ particles/mL of cSEVs and aSEVs were administered for 10 min, followed by 35 min of global ischemia and 120 min of reperfusion and infarct size and perfusion pressure was determined. The results show that cSEVs significantly reduced the infarct size, but this effect was lost with aSEVs (Figure 3A). The administration of aSEVs had no effect in the perfusion pressure at the end of reperfusion (Figure 3B). Collectively, these results suggest that a pro-inflammatory endothelial phenotype does not alter the production of SEVs but can impair their cardioprotective effect.

## Discussion

Our data shows that induction of endothelial activation does not affect the production SEVs but can impair their function in the setting of I/R injury. Unlike our findings, previous studies have shown that stimulation with TNF-α for 24 h increases the production of SEVs in HUVECs, as well as other cell types (Li et al., 2019). This discrepancy may be explained by the methodology employed to measure SEVs concentration. The study by Li. et al. (2019) used μg of proteins to evaluate SEVs production (Li et al., 2019), whereas we used Nanoparticle Tracking Analysis, suggesting the release of multiple factors by endothelial cells upon stimulation with TNF-α does not necessarily correlate with increased SEVs production. However, longer incubation periods or higher concentrations of TNF-α may elicit higher release of SEVs, but future studies may address this possibility appropriately. Indeed, while we did not detect changes in the production of SEVs in response to TNF-α, it’s important to consider that we only incubated for 24 h and thus, an effect at 48 h cannot be ruled out. There are other physiological scenarios in which production of SEVs may be altered. For instance, endothelial cells undergoing senescence, which is a state associated with permanent cell cycle arrest and a pro-inflammatory phenotype (Bloom et al., 2023), have been described to increase the production of functional SEVs (Riquelme et al., 2020). In contrast, our study showed that endothelial activation induced by TNF-α does not affect the production of SEVs, but their function is lost when their production occurs in a pro-inflammatory environment.

To evaluate the function of endothelial SEVs, we chose the assessment of its effect in the context of cardiac I/R injury. The cardioprotective effect of SEVs has been evaluated before. Previous research has shown that plasma SEVs isolated from adult rats can reduce infarct size in *ex vivo* and *in vivo* experimental models (Giricz et al., 2014; Vicencio et al., 2015; Minghua et al., 2018; Wang et al., 2021). Moreover, several studies have demonstrated that SEVs from different cell types can confer protection against cardiac I/R injury (Gallet et al., 2017; Jiang et al., 2018; Maring et al., 2019; Takov et al., 2020). Endothelial SEVs have also been described to reduce cell death after hypoxia/reoxygenation in adult rat cardiomyocytes (Davidson et al., 2018a; Davidson et al., 2018b). However, to our knowledge, this is the first study showing that HUVEC-derived SEVs under baseline conditions can reduce infarct size in *ex vivo* I/R performed in mice hearts. We chose a concentration of 1 × 10^8 because previous work suggests that this concentration of endothelial SEVs may reduce Hypoxia/Reoxygenation-induced cardiomyocyte death (Davidson et al., 2018b). Moreover, this concentration was also reported to reduce cell death using plasma SEVs in the context of I/R injury (Vicencio et al., 2015). However, we cannot discard increased protection with higher concentrations, since HUVEC SEVs have been shown to exert concentration-dependent effects (Riquelme et al., 2020). In addition, we chose 10 min of pre-treatment with SEVs, because it has been previously reported that plasma SEVs can activate the cardioprotective protein Hsp27 at 2, 5 and 15 min (Vicencio et al., 2015). Moreover, these vesicles were administered 15 min before *ex vivo* I/R injury, suggesting a rapid post-translational effect (Vicencio et al., 2015). Moreover, we did not include a washout period between the end of the administration of SEVs and the beginning of global I/R injury, which means that SEVs may have been present in the microcirculation during ischemia. This may constitute a limitation to fully explore the pre-conditioning potential of endothelial SEVs. In addition, administration at the onset of reperfusion may have higher clinical value, but our study was mainly focused in establishing proof-of-concept regarding the effects of a pro-inflammatory state of endothelial cells in the production of functional SEVs that can protect from I/R injury.

The perfusion pressure at the end of reperfusion appears to be high in hearts treated with aSEVs, although we did not observe any statistical difference. While the reason for this effect is unknown, it may be speculated that these nanovesicles may impair vasodilation or elicit hypercontracture of the myocardium, but we do not show evidence to support or test these possibilities.

The translational potential of HUVEC SEVs was demonstrated in a human heart-on-chip subjected to simulated I/R injury (Yadid et al., 2020). Pursuing potential mechanisms, this study also established that endothelial SEVs carry proteins associated with redox balance, metabolism, and calcium handling (Yadid et al., 2020). Plasma SEVs have been found to carry the cardioprotective protein HSP70 (Vicencio et al., 2015), but this protein was undetected in HUVEC SEVs (Davidson et al., 2018a). Furthermore, another study showed that the protective effect of HUVEC-derived SEVs against Hypoxia/Reoxygenation-induced cell death was prevented by inhibition of ERK1/2 (Davidson et al., 2018b), but this observation needs to be confirmed with a more thorough assessment of the activation of this protein, such as evaluating its phosphorylation, as well as a more thorough and solid cell death assessment. Regarding the loss of the cardioprotection under pro-inflammatory conditions, a change in the cargo of endothelial SEVs may be the likely cause of this phenomenon, but whether a pro-inflammatory signal decreases the content of cardioprotective proteins in the nanosized vesicles produced by endothelial cells, remains to be thoroughly addressed.

It has been previously hypothesized that inflammation may impair the effect of cardioprotective strategies, such as ischemic preconditioning (Wojciechowska et al., 2015). Thus, our study not only expands our knowledge regarding the biology of endothelial SEVs, but also supports the idea that inflammation may be a relevant variable to account for in the context of cardioprotection (Wojciechowska et al., 2015). Our results need to be interpreted cautiously. While we show a preliminary proof-of-concept, some of our experiments had limited sample size and/or high variability, suggesting part of this study may be underpowered. Therefore, our findings, although preliminary, reveal that control HUVEC SEVs can reduce the infarct size, but this effect is lost when these nanovesicles are produced by endothelial cells in a pro-inflammatory state, highlighting the relevance of the physiological state of endothelial cells regarding their biological functions.

## Data Availability

The raw data supporting the conclusions of this article will be made available by the authors, without undue reservation.
